# A Classification for Gastric Outlet Obstruction in Childhood: Extending Beyond Infantile Hypertrophic Pyloric Stenosis

**DOI:** 10.5152/tjg.2024.23202

**Published:** 2024-03-01

**Authors:** Ödül Eğritaş Gürkan, Hakan Öztürk, Cem Kaya, Neslihan Gürcan Kaya, Neslihan Ekşi Bozbulut, Ayşe Can, Kamercan Ceylan, Aysel Ünlüsoy Aksu, Demet Teker Düztaş, Sinan Sarı, Buket Dalgıç, Alparslan Kapısız, Demet Coşkun, Gökcen Emmez, Gözde İnan, İsmail Akdulum, Hasan Kutluk Pampal, Nuray Camgoz Eryılmaz, Selin Erel, Volkan Şıvgın, Ercan Yıldırım, Okan Ermiş, İrfan Güngör, Gülay Kip, Nurdan Bedirli, Berrin Işık, İbrahim Onur Özen, Yusuf Hakan Çavuşoğlu, Ramazan Karabulut, Zafer Türkyılmaz, Kaan Sönmez

**Affiliations:** 1 Department of Pediatric Gastroenterology, Gazi University School of Medicine, Ankara, Turkey; 2 Department of Pediatric Surgery, Gazi University School of Medicine, Ankara, Turkey; 3 Department of Anesthesiology, Gazi University School of Medicine, Ankara, Turkey; 4 Department of Pediatric Radiology, Gazi University School of Medicine, Ankara, Turkey

**Keywords:** Gastric outlet obstruction, children, eosinophilic gastroenteritis, antral web

## Abstract

**Background/Aims:**

Gastric outlet obstruction (GOO) is a rare condition in childhood, with the exception of infantile hypertrophic pyloric stenosis (IHPS). However, no classification exists from a pediatric gastroenterologist’s perspective.

**Materials and Methods:**

The patients with a diagnosis of GOO between 2009 and 2020 were reviewed retrospectively. We classified the patients according to GOO: presence of clinical findings accompanied by radiological and/or endoscopic findings; clinical status: intractable nonbilious postprandial vomiting alone or with abdominal pain, early satiety, weight loss, postprandial abdominal distension, and malnutrition; radiology: delayed gastric emptying and dilated stomach; endoscopy: nonbilious gastric contents after 6-8 hours of emptying and/or failed pyloric intubation; physical examination: visible gastric peristalsis.

**Results:**

A total of 30 GOO patients (15 patients with IHPS, 1 patient with annular pancreas, 4 patients with gastric volvulus, 2 patients with duodenal atresia, 2 patients with antral web, 1 patient with late-onset hypertrophic pyloric stenosis (LHPS) had surgical treatment, and remaining 5 patients had medical treatment) were enrolled to the study. The median age was 8 months (range: 3 months-16 years), and 14 patients were female. Mitochondrial disorders, LHPS, metabolic disorders, and eosinophilic gastrointestinal system diseases were added to Sharma’s GOO classification, and the classification has been expanded.

**Conclusion:**

This is the first and largest study of GOO in children. From the perspective of pediatric gastroenterology, new diseases will be addressed, and definitions will be highlighted with our classification for GOO in childhood.

**Main Points**

- Gastric outlet obstruction (GOO) is a rare condition in childhood, with the exception of infantile hypertrophic pyloric stenosis.
- A large number of diseases causing GOO have been described in the last decade.
- A revised classification from a pediatric gastroenterologist’s perspective is presented, and it will serve as a guide for differential diagnosis.

## Introduction

Gastric outlet obstruction (GOO) is a clinical syndrome comprising a broad spectrum of conditions that prevent the passage of gastric content into the duodenum. It is caused by mechanical obstructions or motility disorders. If there is a mechanical obstruction, whether extrinsic or intrinsic, it could be located in the distal stomach (prepyloric area), pyloric channel, or duodenum, especially in the first part. It is a common disorder. It occurs in 2-5 per 1000 infants and children annually. However, excluding patients with infantile hypertrophic pyloric stenosis (IHPS), the incidence of GOO in children decreases to 1 in 100 000 live births.^1-6^

Gastric contents could not pass through the duodenum in patients with GOO. Delayed gastric emptying and dilated stomach are observed radiologically, and patients suffer from abdominal pain, nonbilious postprandial vomiting, early satiety, weight loss, or malnutrition. In 2008, Dr Kamlesh Kumar Sharma, a pediatric surgeon, and colleagues reported the classification of GOO.^7^ Subsequently, a few colleagues reported classifications for GOO based on their approaches and experiences. The classifications in the last decade are simple and categorize GOO into benign or malignant groups.^8-11^ Considering the mechanism of some disorders that cause GOO in childhood, it would not be suitable to divide them into 2 groups. The subgroups of GOO definition used today are unclear. 

The aim of this study was to highlight the points that need to be considered in differential diagnosis of GOO, to clarify subgroup definitions, and to reach a final diagnosis from the perspective of pediatric gastroenterologists.

## Materials and Methods

We retrospectively reviewed pediatric gastroenterology and pediatric surgery outpatient and inpatient records, pediatric surgery operating rooms, and pediatric endoscopy unit archives between 2009 and 2020. The files of the patients diagnosed with GOO were examined in detail.

Gastric outlet obstruction in the presence of clinical findings accompanied by radiological and/or endoscopic findings. Intractable nonbilious postprandial vomiting alone or with abdominal pain, early satiety, weight loss, postprandial abdominal distension, malnutrition, and visible gastric peristalsis were noted as clinical findings. Radiological delayed gastric emptying and dilated-size stomach images were checked. Endoscopic findings as nonbilious gastric contents after 6-8 hours of emptying and/or failed pyloric intubation presence were gathered from the files of GOO patients.

Related terminology of GOO in the literature are listed below:

### Infantile Hypertrophic Pyloric Stenosis 

A pyloric thickness of ≥3 mm and a length of ≥15 mm in the infant period.

### Late-Onset Hypertrophic Pyloric Stenosis

The thickness of the pyloric muscle is expected to be greater than the age value and greater than 15 mm in length.

### Primary Acquired GOO (Late-Onset Primary Acquired Gastric Outlet Obstruction, Pyloric Achalasia, Nonhypertrophic Pyloric Stenosis, and Jodhpur disease)

Length of the pyloric ring is normal or long (more than 15 mm) without hypertrophy.

### Ethical Committee Approval

The study protocol was approved by the Gazi University School of Medicine Local Committee of Ethics for Medical Research (No. 249-13, November 7, 2022). Written informed consent was obtained from parents.

## Results

The number of patients admitted to the Pediatric Gastroenterology and Pediatric Surgery outpatient clinics was 75 795 and 73 294, respectively, while the number of hospitalized patients was 2873 and 35 431, respectively. Out of them, 25 061 patients underwent surgery. Among them, 15 patients with IHPS, 1 patient with annular pancreas, 4 patients with gastric volvulus, 2 patients with duodenal atresia, 2 patients with antral web, and 1 patient with late-onset hypertrophic pyloric stenosis (LHPS) underwent surgery, while 5 patients were treated medically. The clinical characteristics, diagnostic tools, and treatment of patients with gastric outlet obstruction, except for IHPS, are presented in Table 1. In our study, there were 15 patients with IHPS. Endoscopy was not performed for any of these patients. All of the IHPS patients were diagnosed with USG and underwent surgery. The age of the operated patients ranged from 25 days to 5 months, with a male dominance. Except for IHPS, 10 out of 15 patients with GOO had benefits from surgery, and 5 patients had medical treatment (Table 1).

## Discussion

This is the first and largest study of GOO during childhood from a pediatric gastroenterologist’s perspective in the literature. Considering the cases reported in recent years and unclear subgroup definitions, we believe that new diseases will be addressed and definitions will be highlighted in this new classification, which will also serve as a guide for differential diagnosis.

According to our classification, the first group is perhaps the best-known group among pediatric surgeons and gastroenterologists. It includes diseases that require rapid differential diagnosis and intervention. Considering causes such as annular pancreas and gastric volvulus, we found it appropriate to change this group to “congenital intrinsic and extrinsic obstruction of antrum, pylorus, and duodenal bulb” (Table 2).

Group 2 is formed by IHPS and should be mentioned as a separate group, as it is the most common cause of GOO in childhood.^12^ Infantile hypertrophic pyloric stenosis accounts for more than 90% of GOO cases and manifests within 3-10 weeks following birth.^13^ A pyloric thickness of 3 mm or higher and a length of 15 mm or higher on ultrasonography are widely accepted as a diagnostic criteria for IHPS.^14,15^ It is certain that pyloric muscle thickness and length directly correlate with age and weight. The specificity and sensitivity of ultrasound in diagnosing IHPS, in the hands of experienced pediatric radiologists, are very high at 98% and 100%, respectively.^16^ In our study, there were 15 patients with IHPS. Endoscopy was not performed for any patient. All of the patients were diagnosed with USG and underwent surgery. The age of the operated patients in our study ranged from 25 days to 5 months, with a male dominance.

Late-onset hypertrophic pyloric stenosis is acquired during childhood. It has an unknown etiology and shows a late onset. We placed LHPS in group 2b unlike other definitions. The thickness of the pyloric duct is accepted as normal up to 3 mm for the newborn and infantile periods, whereas this thickness limit extends up to 8 mm for adults. Therefore, for any infantile period after the third month or for the older childhood period, pyloric thickness above the age-appropriate limits indicates pyloric hypertrophy. Longitudinal measurements of pyloric ring values, varying according to age or weight in the childhood age group, have not been described in the literature. For the diagnosis of LHPS, the thickness of the pyloric muscle is expected to be greater than the age-appropriate value and greater than 15 mm in length.^14,15^ Symptoms can occur over months to years.^17-20^ A few case reports have reviewed the literature on LHPS. However, in some of these cases, primary acquired gastric outlet obstruction, namely Jodhpur disease, was reported as LHPS.^7,21^ To clarify the definitions for clinicians, whether infantile or LHPS, the thickness and length of the pyloric ring should be greater than age- and weight-specific ranges. Thus, both criteria should be met. In our series, an 8-month-old patient presented with postprandial vomiting, abdominal distension, and poor weight gain. Endoscopy revealed a narrowed pyloric ring, and the endoscope was inserted into the second part of the duodenum. The process of passing through the pylorus provided the feeling of a long tunnel instead of a ring. In USG, the pyloric ring was noted as thick for age (4 mm), and the pyloric length was noted as excessive compared to the month, but the length was not specified by the radiologist.

Secondary acquired GOO causes are categorized as group 3, and many new diseases are gradually being added to this group with advanced endoscopic and metabolic–genetic examinations. 

**Figure 1.**
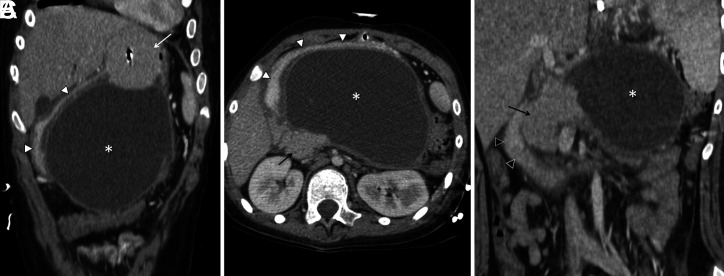
CT images of the patient diagnosed with gastric outlet obstruction and pancreatitis. On contrast-enhanced axial (B) and coronal reformatted (A, C) abdomen CT images, a lobulated, contoured, thin-walled cystic lesion (*) measuring 135 × 88 × 130 mm in the widest part and involving the body and tail of the pancreas is seen. Gastric lumen (white arrowheads) and PEG balloon (white arrow) can be distinguished superior to the cyst, and the pancreatic head and neck (black arrow) can be seen in the lateral aspect of the cyst. On the lateral side of the pancreatic head, we can also discern the natural course of the second and third portions of the duodenum (black arrowheads). CT, computed tomography; PEG, percutaneous endoscopic gastrostomy.

Two patients who were followed up for hypoxic ischemic encephalopathy had severe reflux disease; thus, gastrostomy was deemed appropriate accompanied with reflux surgery. In our series, 2 patients (13-year-old male and 5-month-old female) developed nonbilious vomiting and abdominal distension following bolus feeding during follow-up after gastrostomy. Patients underwent endoscopy after their metabolic conditions improved and infectious causes were excluded. The gastrostomy tubes were seen close to the pylorus, and hyperemia and edema were noted in the surrounding mucosa. Two patients were diagnosed with GOO and hospitalized. Their amylase and lipase values were above 1000 IU/L. Embedded percutaneous endoscopic gastrostomy (PEG) catheters placed in front of the pyloric ring, compressing the pancreas, were noted on CT images of both patients (Figure 1A-C). Pancreatitis and obstruction regressed following the removal of the PEG catheters. 

Apart from eosinophilic esophagitis, eosinophilic gastrointestinal system diseases (EGIDs) are rare in childhood and may present in various forms.^22^ Among them, eosinophilic enteritis is quite interesting and causes cholecystitis and pancreatitis, depending on eosinophilic infiltration in the duodenum. A 15-year-old female patient with EGIDs presenting with GOO for the first time in the pediatric age group was reported in the 4-person adult case series of Jadhav et al^23^ in 2020. Eosinophilic infiltration and inflammation in the duodenum and antrum may cause cholecystitis and pancreatitis. Eosinophilic inflammation in the prepyloric and postpyloric regions may cause GOO via a similar mechanism. In our series, a 6-year-old patient was evaluated for complaints of nonbilious vomiting and chronic recurrent abdominal pain after feeding. Complete blood count revealed 6% eosinophilia. Abdominal USG revealed dilated gastric distension and an elevated thickness of the bowel wall. During endoscopy, gastric distension was noted and the stomach was full of food residue. The gastric contents were removed with nasogastric tube insertion, and after 48 hours of fasting, less food residue was observed in the control endoscopy. The pylorus ring was narrowed, and the endoscope could barely pass into the duodenum. Biopsy samples taken from the prepyloric region and duodenum were compatible with eosinophilic gastritis and eosinophilic enteritis. Our patient presented with GOO and was diagnosed with eosinophilic gastroenteritis. Complaints were fully controlled with steroid and proton pump inhibitor (PPI) treatment.

**Figure 2.**
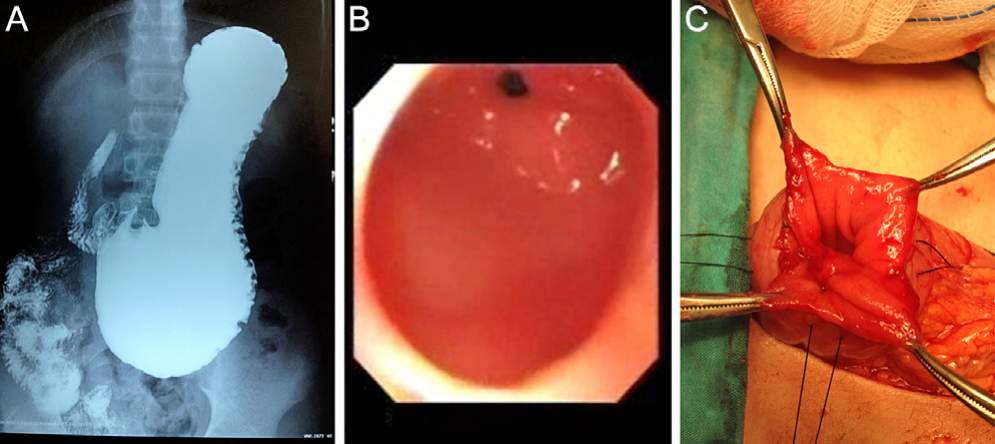
Direct x-ray, endoscopic, and intraoperative images of the antral web case presented with gastric outlet obstruction.

There were 2 antral web cases in our study. These are the first endoscopically diagnosed cases in the literature. Our first patient was 12 years of age and was followed up with cyclic vomiting. The procedure could not be terminated during the first endoscopy because the stomach was full. Secondary endoscopy revealed a narrow pyloric ring and erosive gastritis. The duodenum was not intubated. The second patient was diagnosed with *Helicobacter pylori* gastritis. Antibiotic and PPI treatment were initiated. A dilated stomach was observed in the radiological contrast series. The narrowed pylorus was dilated with a balloon. His symptoms were relieved after balloon dilatation. During 12 consecutive dilatation procedures, a true pyloric ring was observed beyond the enlarged ring, which was considered a pyloric ring. The antral web was surgically resected (Figure 2A-C).

Primary acquired gastric outlet syndrome is a disease with which pediatric gastroenterologists are not familiar. Since no definition can fully explain its clinical signs and physiology, it has found its place with different names in the literature, such as primary acquired gastric outlet obstruction, late-onset primary acquired gastric outlet obstruction, pyloric achalasia, nonhypertrophic pyloric stenosis, or Jodhpur disease. Pyloric achalasia or nonhypertrophic pyloric stenosis refers to cases in which the pyloric length is >15 mm without hypertrophy. The existence of different names is because of the fact that no one name can adequately represent the underlying etiology or clinical signs. 

In 2008, Sharma et al^7^ reported their own cases on the subject and provided examples of cases with Jodhpur disease that were mistakenly presented as LHPS. There was 1 case of LHPS in our study. Differential diagnosis for Jodhpur disease was based on pyloric length and muscle thickness criteria according to age. Unchanging and common findings in this group of diseases include nonbilious vomiting after feeding, abdominal distension and/or abdominal pain, radiologically delayed gastric emptying, gastric distension, and improvement with surgical treatment. In imaging methods, the length of the pyloric ring may be normal or long (>15 mm) without hypertrophy. Since there is no pathology regarding the length of the pylorus radiologically and the appearance with the naked eye is normal during the operation, the benefit from surgical operations is an indication that the pyloric ring is abnormal in terms of function. 

Our study had several limitations: It was a single-centered study and contained limited number of patients. Nevertheless, it is one of the largest studies in the pediatric age group and brings clarity to definitions of the terms used in the literature.

**Figure 3.**
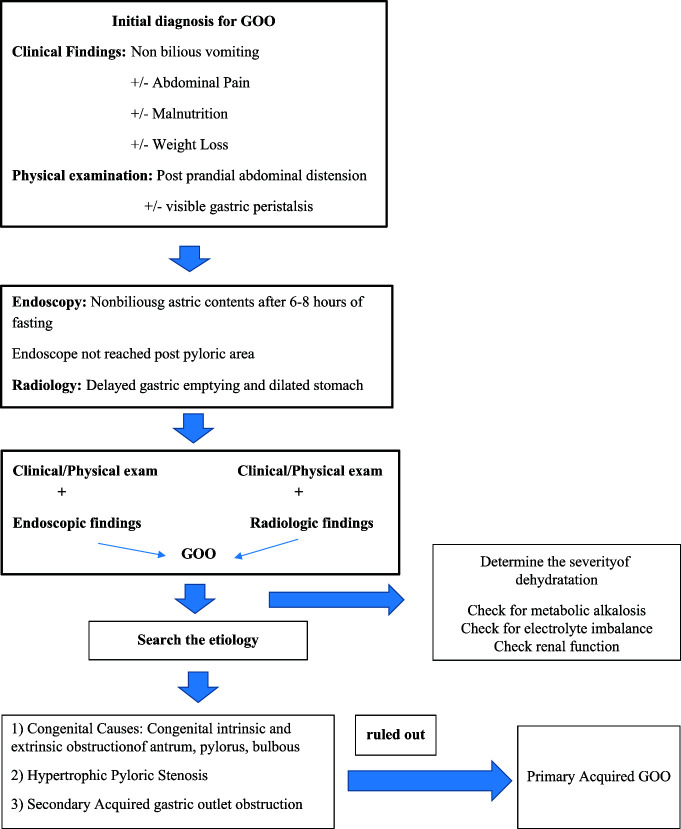
Work chart suggested in the diagnosis and differential diagnosis of gastric outlet obstruction. GOO, gastric outlet obstruction.

As a result, GOO, other than IHPS, is a rare condition in childhood. Perhaps the most important part of the initial evaluation of a GOO patient for etiology is to have foresight about whether patients will benefit from surgical treatment or medically noninvasive procedures (Figure 3). We may encounter new diseases to be added to the secondary acquired GOO group daily. Improved endoscopic techniques would be more useful for diagnosing GOO in the pediatric age group.

This is the first and largest study on GOO during childhood from the perspective of a pediatric gastroenterologist to be reported in the literature. It is aimed to clearly define and review all the terminology related to GOO. Definitions related to GOO in the pediatric age group are very important. Two diseases are often confused with each other and their terminologies are used interchangeably in case reports: “late-onset hypertrophic pyloric stenosis (LHPS)” and “primary acquired gastric outlet obstruction (late-onset primary acquired gastric outlet obstruction, pyloric achalasia, nonhypertrophic pyloric stenosis, Jodhpur disease).”

## Figures and Tables

**Table 1.** Clinical Characteristics, Diagnostic Tools, and Treatment of Patients with Gastric Outlet Obstruction, Except Infantile Hypertrophic Pyloric Stenosis

Patient Number	Etiology of GOO	Accompanying Disease	Sex	Age of Diagnosis (Months)	Clinical Status	Endoscopy	Radiology	Treatment
1	PEG	Cerebral palsy	Female	6	Vomiting + abdominal distension	PEG balloon causing obstruction in the prepyloric region	–	Removal of PEG + PPI treatment
2	PEG + pancreatitis	Cerebral palsy	Male	156	Vomiting	PEG balloon causing obstruction in the prepyloric region	+	Removal of PEG + PPI, octreotide treatment
3	HP gastritis	Down syndrome	Female	192	Vomiting + abdominal pain	The pyloric ring is hyperemic and edematous, too narrow to allow the scope to pass	UGIS: gastroptosis and distended stomach	HP eradication treatment
4	Annular pancreas	–	Female	15	Vomiting	–	UGIS: delayed gastric emptying, gastric distension CT: annular pancreas	Surgery
5,6,7,8	Gastric volvulus	–	Female [1] Male [3]	3 4, 5, 18	Vomiting	–	UGIS: organoaxial volvulus	Surgery 3
9,10	Duodenal atresia	–	Female [2]	3, 4	Vomiting	–	UGIS: compatible with duodenal atresia	Surgery
11	Antral web	Cyclic vomiting syndrome	Male	144	Vomiting + abdominal pain	Antral web	UGIS: gastric distension + delayed gastric emptying	Balloon dilatation surgery
12	Antral web	-	Male	18	Vomiting	Antral web	UGIS: compatible with antral web	Surgery
13	Crohn’s disease	Cyclic vomiting syndrome 3	Female	16	Vomiting + abdominal pain	Large ulcers covered with white exudate in prepyloric region. The pyloric configuration is impaired and does not allow the passage of the scope.	–	Immunosuppressive treatment
14	Late-onset HPS	–	Female	8	Vomiting + abdominal distension	Narrowed pyloric ring	USG appearance compatible with HPS	Surgery
15	Eosinophilic gastroenteritis	–	Male	4	Vomiting + abdominal pain	Erosive pangastritis, bulbitis, and the endoscope did not advance to the second part of the duodenum	Gastric distension	PPI + diet

CT, computed tomography; HP, *Helicobacter pylori;*HPS, hypertrophic pyloric stenosis; PEG, percutaneous endoscopic gastrostomy; PPI, proton pump inhibitor; UGIS, upper gastrointestinal series.

**Table 2.** Classification for Gastric Outlet Obstruction in Childhood

Group	Cause
1: Congenital intrinsic and extrinsic obstruction of the antrum, pylorus, and bulbous	a) Aplasia b) Atresia c) Diaphragm and web d) Luminal obstruction (e.g., mucosal valves, heterotrophic pancreas) d) Annular pancreas
2: a) IHPS (congenital or infantile hypertrophic pyloric stenosis) b) Late-onset hypertrophic pyloric stenosis	3
3) Acquired	**a) Primary** (I) Acquired gastric outlet obstruction during infancy and childhood (primary acquired gastric outlet obstruction, late-onset primary acquired gastric outlet obstruction, pyloric achalasia, nonhypertrophic pyloric stenosis, Jodhpur disease)
	**b) Secondary** (I) Acid peptic disease (II) Neoplasm (III) Chemical injury (IV) EGIDs (V) Crohn’s disease (VI) Polyp (VII) Bezoars (VIII) Infectious causes (Tbc, CMV) (IX) Amyloidosis (X) Pancreatitis (XI) PEG complication (XII) Metabolic disorders (XIII) Mitochondrial disorders

EGIDs, eosinophilic gastrointestinal system diseases; IHPS, infantile hypertrophic pyloric stenosis; PEG, percutaneous endoscopic gastrostomy.
